# Application of Recombinant Rabies Virus to *Xenopus* Tadpole Brain

**DOI:** 10.1523/ENEURO.0477-20.2021

**Published:** 2021-07-03

**Authors:** Regina L. Faulkner, Nicholas R. Wall, Edward M. Callaway, Hollis T. Cline

**Affiliations:** 1 Neuroscience Department and The Dorris Neuroscience Center, The Scripps Research Institute, La Jolla, CA 92037; 2 The Salk Institute for Biological Sciences, La Jolla, CA 92037

**Keywords:** recombinant rabies virus, retrograde labeling, *Xenopus*, optic tectum, mesoscale connectomics

## Abstract

The *Xenopus laevis* experimental system has provided significant insight into the development and plasticity of neural circuits. *Xenopus* neuroscience research would be enhanced by additional tools to study neural circuit structure and function. Rabies viruses are powerful tools to label and manipulate neural circuits and have been widely used to study mesoscale connectomics. Whether rabies virus can be used to transduce neurons and express transgenes in *Xenopus* has not been systematically investigated. Glycoprotein-deleted rabies virus transduces neurons at the axon terminal and retrogradely labels their cell bodies. We show that glycoprotein-deleted rabies virus infects local and projection neurons in the *Xenopus* tadpole when directly injected into brain tissue. Pseudotyping glycoprotein-deleted rabies with EnvA restricts infection to cells with exogenous expression of the EnvA receptor, TVA. EnvA pseudotyped virus specifically infects tadpole neurons with promoter-driven expression of TVA, demonstrating its utility to label targeted neuronal populations. Neuronal cell types are defined by a combination of features including anatomic location, expression of genetic markers, axon projection sites, morphology, and physiological properties. We show that driving TVA expression in one hemisphere and injecting EnvA pseudotyped virus into the contralateral hemisphere, retrogradely labels neurons defined by cell body location and axon projection site. Using this approach, rabies can be used to identify cell types in *Xenopus* brain and simultaneously to express transgenes which enable monitoring or manipulation of neuronal activity. This makes rabies a valuable tool to study the structure and function of neural circuits in *Xenopus*.

## Significance Statement

Studies in *Xenopus* have contributed a great deal to our understanding of brain circuit development and plasticity, regeneration, and hormonal regulation of behavior and metamorphosis. Here, we show that recombinant rabies virus transduces neurons in the *Xenopus* tadpole, enlarging the toolbox that can be applied to studying *Xenopus* brain. Rabies can be used for retrograde labeling and expression of a broad range of transgenes including fluorescent proteins for anatomic tracing and studying neuronal morphology, voltage or calcium indicators to visualize neuronal activity, and photosensitive or chemosensitive channels to control neuronal activity. The versatility of these tools enables diverse experiments to analyze and manipulate *Xenopus* brain structure and function, including mesoscale connectivity.

## Introduction

Studies in *Xenopus* have yielded insights into research including neural development, plasticity, and regeneration. Progress in these areas of investigation would be facilitated by additional tools to study the structure and function of defined cell types within the *Xenopus* brain. Viruses are an excellent tool for gene manipulation *in vivo* as they can be targeted to specific cell types with high spatiotemporal precision. Viruses can deliver a broad range of transgenes including fluorescent proteins for anatomic tracing and studying neuronal morphology, voltage or calcium indicators to visualize neuronal activity, and photosensitive or chemosensitive channels to control neuronal activity (Osakada et al., 2011; Callaway and Luo, 2015). In *Xenopus*, viral tools are limited. Neither adeno-associated virus (AAV) nor lentivirus transduce brain cells in *Xenopus* (Yamaguchi et al., 2018). Vaccinia virus and vesicular stomatitis virus (VSV) have been used for gene expression in *Xenopus* (Wu et al., 1995; Mundell et al., 2015; Yamaguchi et al., 2018), but expression from Vaccinia is transient and VSV transduction is inefficient (A. Yamaguchi, personal communication). Therefore, we investigated applications of rabies virus in *Xenopus*.

Recombinant rabies viruses have been widely used to investigate the structure and function of neural circuits. Depending on the viral variant, rabies virus can be used for retrograde neuroanatomical tracing, transsynaptic tracing, and transgene expression in genetically-defined cell types in specific anatomic locations (Ugolini, 2010; Callaway and Luo, 2015; Nassi et al., 2015; Luo et al., 2018). Wild-type rabies virions infect neurons at their axon terminals, are transported retrogradely to the soma, and replicate before spreading to presynaptically connected neurons (Schnell et al., 2010). Deletion of the glycoprotein gene from the rabies genome eliminates the ability of viral replication to produce infectious particles. Complementing glycoprotein (G)-deleted rabies virus with a viral envelope protein generates rabies virions which are infectious and transport retrogradely, but do not infect presynaptically connected neurons (Mebatsion et al., 1996b; Etessami et al., 2000; Wickersham et al., 2007a). G-deleted rabies virus infects neurons at their axon terminals and acts as a retrograde vector for expression of transgenes. When the deleted glycoprotein is supplied exogenously in infected neurons (called “in *trans*”), the viral particles spread monosynaptically to presynaptic neurons (Wickersham et al., 2007b; Stepien et al., 2010). Infection of recombinant rabies can be restricted to genetically-defined neuronal populations by pseudotyping with the envelope glycoprotein for the avian sarcoma and leukosis virus, EnvA. Transfecting genetically defined neuronal populations with the receptor for EnvA, TVA, restricts infection to those cell types (Young et al., 1993; Wickersham et al., 2007b; Marshel et al., 2010; Wall et al., 2010), facilitating cell type identification and study. Neuronal subtypes are defined by a combination of features including cell body location, genetic markers, morphology, axonal projections, and physiological properties (Ecker et al., 2017). EnvA pseudotyped rabies can identify neurons simultaneously defined by cell body location through retrograde tracing, genetic control of TVA expression, and axonal projection pattern by localizing viral injection to specific target locations. These rabies viral variants permit flexible investigation into diverse aspects of neural circuit structure and function.

We examined whether recombinant rabies viruses can be used to express transgenes in *Xenopus laevis* brain. Direct injection of G-deleted rabies virus phenotypically complemented with surface rabies glycoprotein (Conzelmann et al., 1990; Wickersham et al., 2007a) into the tadpole optic tectum infects both local tectal neurons and neurons in contralateral tectum and hindbrain, demonstrating efficient retrograde transport in axons. Previous work has shown that intertectal communication is important for visuomotor behavior in tadpoles (Gambrill et al., 2016). Combining rabies virus injection with *post hoc* immunohistochemistry for GABA demonstrated that both excitatory and inhibitory neurons project axons between the two optic tectal lobes, indicating the utility of rabies to study mesoscale connectivity in *Xenopus*. Expression of rabies glycoprotein in *trans* in infected tectal neurons did not result in transsynaptic spread and presynaptic viral infection. Pseudotyping the virus with EnvA restricted infection to neurons transfected with TVA. Using EnvA pseudotyped virus, we achieved retrograde infection of targeted neuronal populations by driving TVA expression with different promoters in afferent neurons and injecting virus into target areas. These results suggest that recombinant rabies viruses could be used to express transgenes in cells triply defined by cell body location, genetics, and axon projection. This combinatorial labeling approach will help uncover cell types in *Xenopus* brain. When paired with the breadth of rabies viral variants that are available to assay neuronal function, this tool will be very useful for investigating the structure and function of neural circuits in this tractable model system.

## Materials and Methods

### Animals

Albino *X. laevis* tadpoles of either sex were obtained by in-house breeding or purchased from Nasco or *Xenopus* Express (catalog #ATAD, RRID:XEP_Xla200). Tadpoles were reared in 0.1× Steinberg’s solution on a 12/12 h light/dark cycle at 22–23°C unless otherwise stated. Experiments were performed between developmental stages 42–48 (Nieuwkoop and Faber, 1956) and tadpoles were anesthetized in 0.02% tricaine methanesulfonate (MS222, Sigma catalog #A5040) before all procedures. All animal protocols were approved by the Institutional Animal Care and Use Committee of The Scripps Research Institute.

### Preparation and injection of pseudotyped recombinant rabies virus

We used envelope G-deleted rabies virus expressing EGFP (SADΔG-EGFP) phenotypically complemented with its native glycoprotein, B19G, or pseudotyped with the envelope glycoprotein for the avian sarcoma and leukosis virus, EnvA (Wickersham et al., 2007a,b). Viruses were injected at titers between 4.4 × 10^8^ and 1.8 × 10^9^ transducing units (TU)/ml. Viruses were either grown and purified as described in Wickersham et al. (2010) or purchased from the Salk GT3 viral vector core (RRID:SCR_014847). For injection, animals were anesthetized in 0.02% MS222 and virus was pressure injected directly into the optic tectum. Virus was injected into the widest part of the tectal lobe. 0.01% Fast Green dye (Sigma catalog #F7252) was added to viral aliquots before injection for visualization and care was taken to ensure that injected virus did not leak into the brain ventricle.

### Electroporation and plasmid constructs

Optic tectal neurons were transfected with TVA800 to mediate SADΔG-EGFP(EnvA) infection, turboRFP (tRFP) to identify TVA-expressing cells, and B19G to generate infectious virions for possible transsynaptic tracing. The CMV promoter was used to drive expression in all neuron types. The vesicular GABA transporter (VGAT) promoter was used to bias expression toward inhibitory neurons (He et al., 2016). In some experiments expression was amplified using the gal/UAS system (Chae et al., 2002). Plasmids were generated to express each protein of interest individually, or to co-express TVA and tRFP. For co-expression, a bi-directional plasmid was used to drive TVA from a sCMV IE94 enhancer/promoter and tRFP from an independent sCMV promoter. The plasmids used in the study were: CMV::TVA/tRFP (RRID:Addgene_164486), CMV::B19G/tRFP, CMV::B19G (RRID:Addgene_15785), CMV::gal4, VGAT::gal4, UAS::tRFP, UAS::TVA (RRID:Addgene_164487), and UAS::B19G. We transfected neurons using whole-brain or micropipette-mediated electroporation. For whole-brain electroporation, tadpoles were anesthetized in 0.02% MS222, plasmids at a concentration of 1 μg/μl were pressure injected into the brain ventricle, and then platinum electrodes were placed on each side of the midbrain and voltage pulses were applied across the midbrain (Haas et al., 2002). Whole-brain electroporations transfected cells throughout the rostrocaudal extent of the tectum. For micropipette-mediated electroporation, tadpoles were anesthetized in 0.02% MS222, a glass pipette with filament containing 1 μg/μl plasmid DNA was inserted directly into the brain and brief electrical stimulation was delivered by an Axoporator 800A (Molecular Devices; Bestman et al., 2006). Micropipette-mediated electroporation transfects one or a few cells near the tip of the micropipette.

### *In vivo* imaging

Stage 42–48 tadpoles were injected with SADΔG-EGFP(B19G) virus or electroporated with expression constructs and injected with SADΔG-EGFP(EnvA) virus 4 d later. One to 7 d following viral injection, animals were anesthetized in 0.02% MS222 and imaged on a PerkinElmer Ultraview Vox spinning disk confocal microscope with a 25× Nikon water-immersion objective lens (1.1 NA). Z-stacks through the entire optic tectum were collected. In some experiments, images were acquired in multiple anatomic locations and montages of Z-projections were created manually.

### Visual stimulation

After electroporation and viral injection, tadpoles receiving visual experience were exposed to a simulated motion stimulus consisting of rows of LEDs illuminating in turn (Sin et al., 2002). This visual experience was provided for either (1) 4 h, 2 d before viral injection, or (2) 12 h overnight, the night before viral injection. The remainder of the time, animals were reared on a normal cycle of 12/12 h light/dark.

### Cell culture

Cell lines were used for Western blotting and immunohistochemistry to test exogenous expression of B19G. 293T cells (RRID:CVCL_0063) were grown in 90% DMEM (with 4.5 g/l D-glucose, L-glutamine, sodium pyruvate, and sodium bicarbonate) and 10% fetal bovine serum at 37°C in 5% CO_2_. The XLK-WG cell line is derived from *Xenopus* kidney cells (ATCC catalog #CRL-2527, RRID:CVCL_5655). XLK-WG cells were grown in 60% RPMI 1640 media (with 2 mm L-glutamine, 10 mm HEPES, 1 mm sodium pyruvate, 4.5 g/l glucose, and 1.5 g/l sodium bicarbonate), 20% fetal bovine serum, and 20% distilled water. XLK-WG cells were maintained at 32°C in 5% CO_2_. Cells were transfected with CMV::B19G and α-actin::GFP using calcium phosphate (293T) or Lipofectamine (XLK-WG) and 24–48 h later cells were collected for Western blotting or fixed for immunohistochemistry.

### Western blottings

For Western blottings of exogenously expressed B19G in cell cultures, cells were scraped from the culture plate of untransfected cells or cells 24 h after transfection with CMV::B19G and α-actin::GFP and homogenized in RIPA buffer. The membrane fraction was isolated by centrifugation. Small aliquots were taken to measure protein concentration using the BCA Protein Assay kit (Thermo Scientific catalog #23227). Then, 0.1 volume of sample buffer was added to 1 volume of sample and boiled for 5 min. The same amount of protein was loaded from each condition and was separated on an SDS-polyacrylamide gel and proteins were transferred to a nitrocellulose membrane. Membranes were blocked in 5% milk and 0.05% Tween 20 in TBS and then incubated in 1:500 mouse anti-rabies (Millipore catalog #MAB8727, RRID:AB_571110) primary antibody overnight at 4°C. Blots were rinsed and incubated in 1:500 goat anti-mouse HRP-conjugated secondary (Bio-Rad catalog #172-1011, RRID:AB_11125936) at room temperature. β-Tubulin (1:750, Sigma catalog #T8535, RRID:AB_261795) was used as a loading control. An ECL chemiluminescence kit (Thermo Scientific catalog #32106) was used to visualize labeling.

### Immunohistochemistry

To investigate whether membrane expression of B19G was observed in XLK-WG cells *in vitro*, we performed immunohistochemistry without permeabilization. Two days after transfection with CMV::B19G and α-actin::GFP, cells were fixed in 4% paraformaldehyde for 15 min and blocked in 10% BSA for 45 min at room temperature. Next, coverslips were incubated in 1:2000 mouse anti-rabies (Millipore catalog #MAD8727, RRID:AB_571110) in 3% BSA overnight at 4°C, followed by 45 min in 1:200 donkey anti-mouse Alexa Fluor 647 (ThermoFisher catalog #A-31571, RRID:AB_162542). As a control for primary antibody specificity, immunohistochemistry was also performed on XLK-WG transfected only with α-actin::GFP. Coverslips were mounted in Prolong Diamond Antifade Mountant (ThermoFisher catalog #P36970) and imaged with a Nikon A1R confocal microscope with a 25× Nikon water-immersion objective lens (1.1 NA).

To investigate exogenous expression of B19 glycoprotein *in vivo*, tadpoles were electroporated with CMV::B19G/tRFP and fixed 3–4 d later for immunohistochemistry. Tadpoles were anesthetized with 0.02% MS222, immersed in 4% paraformaldehyde, and fixed using two bouts of microwave fixation at 150 W for 1 min followed by overnight fixation at 4°C. Brains were dissected, embedded in a gelatin-albumin mixture, and sectioned at 40–50 μm on a vibratome. Sections were blocked and permeabilized in 5% normal donkey serum and 1% Triton X-100 for 1 h at room temperature. Then, sections were incubated in 1:500 mouse anti-rabies (Millipore catalog #MAB8727, RRID:AB_571110) for 2 d at 4°C followed by 2 h in 1:200 goat anti-mouse Alexa Fluor 405 (ThermoFisher catalog #A-31553, RRID:AB_221604) or Alexa Fluor 488 (ThermoFisher catalog #A-11001, RRID:AB_2534069) at room temperature. Sections were mounted in Gel mount (Accurate) and imaged with a PerkinElmer Ultraview Vox spinning disk confocal microscope with a 25× Nikon water-immersion objective lens (1.1 NA).

## Results

### Recombinant rabies virus infects *Xenopus* neurons

**Figure 1.**
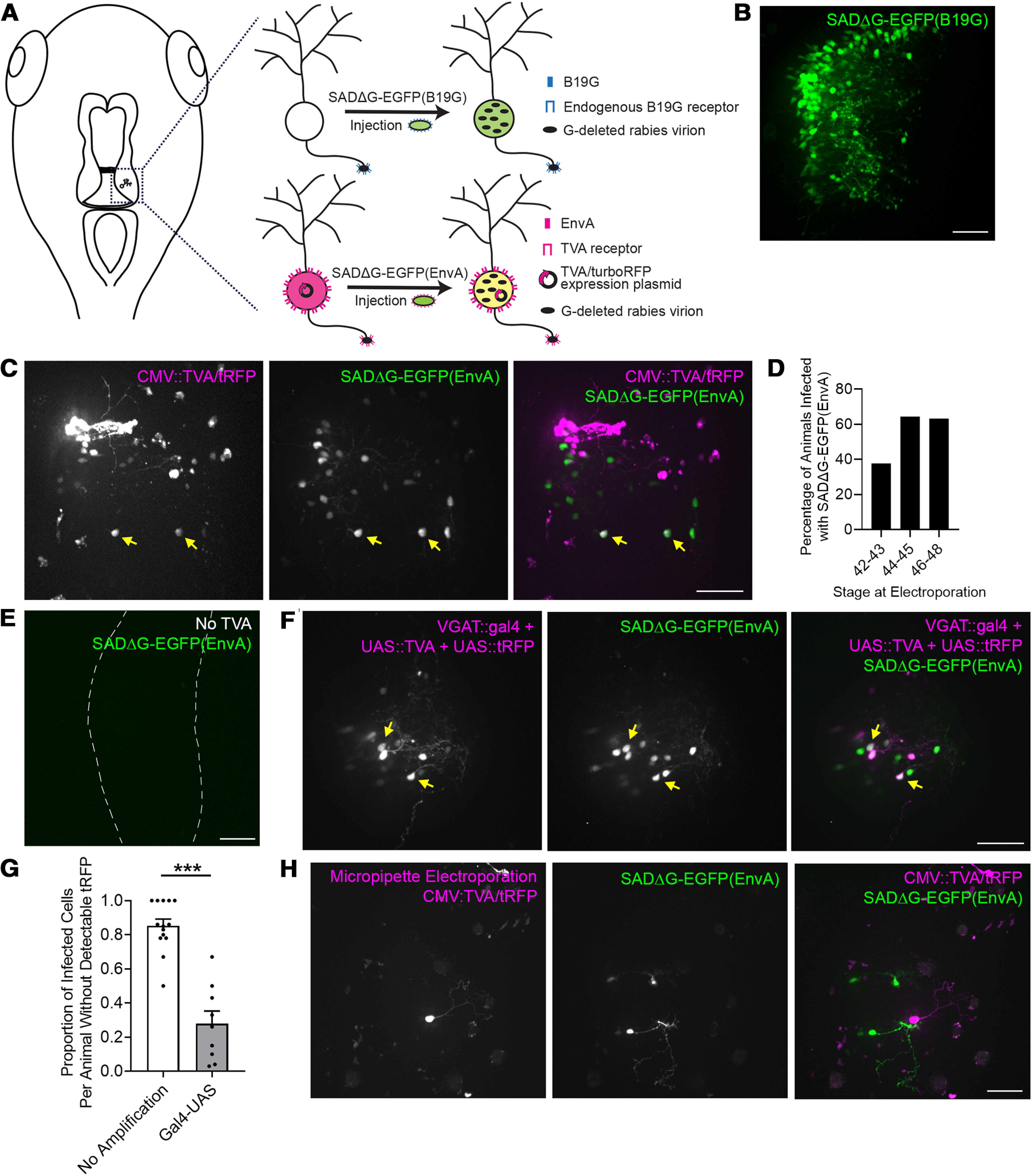
Pseudotyped recombinant rabies virus infects tectal neurons in the *Xenopus* tadpole. ***A***, Schematic of the labeling strategies using recombinant SAD B19 rabies virus which has the glycoprotein deleted and replaced by EGFP (SADΔG-EGFP), rendering it incapable of transneuronal spread. Infection with B19G phenotypically complemented virus (top) relies on endogenous expression of the B19G receptor. Infection with EnvA pseudotyped virus (bottom) requires exogenous expression of its receptor, TVA, before viral injection. Co-labeling TVA-transfected neurons with tRFP allows them to be identified. Viral injections were made in the optic tectum, which is marked by a dashed box in the drawing to the left. ***B***, SADΔG-EGFP(B19G) virus infects tectal neurons. Confocal Z-projection collected *in vivo* through the injected optic tectal lobe shows widespread virally-mediated expression of EGFP in infected neurons. ***C***, SADΔG-EGFP(EnvA) virus infects tectal neurons transfected with TVA. The right optic tectal lobe was transfected with CMV::TVA/tRFP by whole-brain electroporation and injected with SADΔG-EGFP(EnvA) virus 4 d later. Confocal Z-projections collected *in vivo* through the optic tectal lobe electroporated with CMV::TVA/tRFP (magenta) and injected with SADΔG-EGFP(EnvA) virus (green). Neurons which co-express TVA/tRFP and viral EGFP are marked by yellow arrows. The remaining EGFP-expressing neurons lack detectable tRFP expression and are presumably invisible TVA-expressing neurons. ***D***, Viral infection efficiency varies with developmental stage. Tadpoles at stages 42–43 (*n* = 16 tadpoles), 44–45 (*n* = 28 tadpoles), or 46–48 (*n* = 65 tadpoles) were electroporated with CMV::TVA/tRFP using whole-brain electroporation. Four days later, the transfected tectal lobe was injected with SADΔG-EGFP(EnvA) virus. The percentage of animals with EGFP-expressing neurons was highest between stages 44 and 48. ***E***, Infection with SADΔG-EGFP(EnvA) virus requires TVA. Confocal Z-projection collected *in vivo* through an optic tectal lobe injected with SADΔG-EGFP(EnvA) shows no infected neurons in the absence of TVA electroporation. ***F***, SADΔG-EGFP(EnvA) infects tectal neurons transfected with TVA driven by the VGAT promoter. The right optic tectal lobe was transfected with VGAT::gal4, UAS::TVA, and UAS::tRFP by whole-brain electroporation and injected with SADΔG-EGFP(EnvA) virus 4 d later. Confocal Z-projections collected *in vivo* through the optic tectal lobe showing electroporated (magenta) and infected (green) tectal neurons. Neurons which co-express TVA/tRFP and viral EGFP are marked by yellow arrows. The remaining EGFP-expressing neurons lack detectable tRFP expression and are invisible TVA-expressing neurons. ***G***, Quantification of the proportion of invisible TVA cells per animal with and without amplification. Amplifying tRFP expression using the gal4-UAS system decreases the proportion of infected, EGFP^+^ cells that lack detectable tRFP compared with tRFP driven by the CMV promoter without amplification. Data are presented as mean ± SEM overlaid with individual data points (***p < 0.0001, Mann–Whitney test). ***H***, Targeted electroporation of TVA/tRFP does not eliminate invisible TVA-expressing neurons. Micropipette-mediated electroporation was used to limit transfection with TVA/tRFP to one or few neurons in the right optic tectal lobe. Four days later, the electroporated tectal lobe was injected with SADΔG-EGFP(EnvA) virus. Confocal Z-projection collected *in vivo* through the optic tectal lobe shows that EGFP-expressing infected neurons which lack detectable tRFP are still present. Scale bars: 50 μm.

To test whether rabies virus infects neurons in the *Xenopus* tadpole, we used the recombinant rabies virus SADΔG-EGFP in which the glycoprotein from the SAD B19 rabies strain is deleted and replaced with EGFP (Conzelmann et al., 1990; Wickersham et al., 2007a). Amplifying SADΔG-EGFP using complementing cell lines which express an envelope glycoprotein makes infectious viral particles, but the virus generates virions which lack glycoprotein and cannot spread. We used SADΔG-EGFP phenotypically complemented with its native glycoprotein, B19G, or pseudotyped with the envelope glycoprotein for the avian sarcoma and leukosis virus, EnvA (Wickersham et al., 2007a,b). Infection with SADΔG-EGFP(B19G) virus occurs in cells expressing any endogenous B19G receptor. SADΔG-EGFP(EnvA) virus requires expression of the TVA receptor to mediate infection. TVA is found only in birds and requires exogenous expression, permitting experimental control of the cell types infected (Young et al., 1993; Federspiel et al., 1994; Wickersham et al., 2007b). We tested whether SADΔG-EGFP(B19G) and SADΔG-EGFP(EnvA) viruses infect neurons in the *Xenopus* tadpole optic tectum (Fig. 1*A*). Our first goal was to evaluate a variety of conditions to ascertain when viral infection occurred. As an initial metric to screen the degree of infection, we quantified the proportion of animals with EGFP-expressing rabies infected neurons.

SADΔG-EGFP(B19G) virus infects neurons at axon terminals and retrogradely labels infected somata with EGFP (Wickersham et al., 2007a). In stage 46–48 tadpoles, we found that injection of SADΔG-EGFP(B19G) virus directly into one optic tectal lobe resulted in robust EGFP expression near the injection site (*n* = 28/33 tadpoles, 85% infected; Fig. 1*B*), presumably from uptake at local axon terminals. We found that injection of SADΔG-EGFP(B19G) into the ventricle produced widespread infection. Therefore, care was taken to ensure that virus was injected directly into brain tissue without leaking into the ventricle in all experiments. Tadpoles are reared at 22°C and we postulated that this decreased body temperature might lead to decreased efficiency of viral infection compared with warm-blooded vertebrates, like rodents, in which rabies virus has been used extensively. Short term incubations at increased temperature have previously been shown to improve infection efficiency in *Xenopus* with other viruses (Dutton et al., 2009) and with G-deleted rabies in fish (Dohaku et al., 2019). To test whether increasing the rearing temperature increased the proportion of tadpoles infected with rabies virus, we injected animals with SADΔG-EGFP(B19G) virus and incubated them at 26°C or 28°C for 4 h immediately following viral injection and again 24 h later. Tadpoles were housed at 22°C for the remainder of the experiment. Five to 7 d later, we found that 83% of tadpoles (*n* = 10/12 tadpoles) reared continuously at 22°C were infected, while 64% of tadpoles (*n* = 7/11 tadpoles) temporarily incubated at 26–28°C were infected, suggesting that increased temperature did not impact infection rates (*p* = 0.37_a_; Table 1). In the infected animals, the number and brightness of EGFP^+^ neurons did not appear different between the groups. These results demonstrate that at normal rearing temperature, a large majority of tadpoles have infected neurons with robust expression of EGFP following injection of B19G phenotypically complemented rabies virus.

Next, we tested whether TVA expression could be used to mediate infection of SADΔG-EGFP(EnvA) virus in targeted neuronal populations. We transfected tectal neurons with a dual CMV promoter expression plasmid to drive pan-neuronal TVA and tRFP expression (CMV::TVA/tRFP) using whole-brain electroporation (Haas et al., 2002). Four days later, when tRFP expression was strong, we injected SADΔG-EGFP(EnvA) virus directly into the transfected optic tectum. Three days after viral injection we observed a subset of tRFP^+^ neurons were infected with pseudotyped rabies virus, as identified by EGFP expression (*n* = 41/65 tadpoles, 63% infected; Fig. 1*C*). Therefore, we achieved infection and robust EGFP expression in a majority of animals with SADΔG-EGFP(EnvA) virus.

To test whether pseudotyped rabies virus-mediated infection might vary with the developmental stage of the tadpoles, we used whole-brain electroporation to transfect tadpoles ranging from stages 42–48 with CMV::TVA/tRFP and injected virus into the tectum 4 d later. Infection was most efficient when tadpoles were electroporated between stages 44 and 48 (Fig. 1*D*), which are ideal stages for performing studies of development and experience-dependent plasticity in tadpoles *in vivo*.

In mouse, infection with SADΔG-EGFP(EnvA) virus requires exogenous TVA expression and there is no infection in the absence of TVA (Wickersham et al., 2007b; Wall et al., 2010). However, we observed a number of EGFP^+^ cells without detectable expression of TVA/tRFP in these experiments (Fig. 1*C*). To determine whether TVA expression is required for infection with SADΔG-EGFP(EnvA) virus in *Xenopus*, we injected SADΔG-EGFP(EnvA) virus into the tectum of untransfected tadpoles. TVA-expressing animals were also injected with the same viral aliquot as a positive control and animals were imaged using identical imaging parameters 6 d later. While the majority of TVA-expressing tadpoles were infected 6 d after viral injection (*n* = 13/18 tadpoles), we did not observe any EGFP^+^ cells in the absence of TVA (*n* = 0/21 tadpoles; Fig. 1*E*). This result indicates that the virus cannot infect *Xenopus* neurons in the absence of the TVA receptor and suggests that EGFP^+^ cells that we observed in TVA/tRFP transfected animals express a low level of TVA which is sufficient to mediate infection, but the tRFP is below detection threshold. These so-called “invisible TVA” neurons have been noted in other studies, in particular when using Cre-dependent gene expression which can have some leakage, because of the very sensitive interaction between EnvA and TVA (Callaway and Luo, 2015; Hafner et al., 2019; Lavin et al., 2020). Increasing expression of co-transfected fluorescent proteins in TVA-expressing neurons and reducing the affinity of EnvA for TVA by mutating TVA have been used to reduce the number of invisible TVA cells (Miyamichi et al., 2013).

To assess whether increasing tRFP expression would decrease the number of invisible TVA cells, we used the gal4-UAS bipartite transcriptional system to amplify gene expression (Chae et al., 2002; Hirsch et al., 2002). In addition to using the CMV promoter, we drove TVA and tRFP expression using the VGAT promoter, which has previously been shown to increase transfection of inhibitory neurons in the tectum (He et al., 2016). The proportion of excitatory:inhibitory neurons in the optic tectum is 70:30 (Miraucourt et al., 2012). Using *post hoc* immunohistochemistry for GABA, it has been demonstrated that the VGAT promoter increases expression in inhibitory neurons so that the transfected population is 50:50 excitatory:inhibitory (He et al., 2016). Co-electroporation of VGAT::gal4 or CMV::gal4 with UAS::TVA, and UAS::tRFP into the tectum, followed by injection of SADΔG-EGFP(EnvA) virus 4 d later resulted in infection in the majority of tadpoles (*n* = 25 tadpoles, 60% infected; Fig. 1*F*). The proportion of tadpoles with infected neurons was similar between the two promoters (CMV::gal4: *n* = 3/4 tadpoles; VGAT::gal4: *n* = 12/21 tadpoles; *p* = 0.63_b_; Table 1). We observed a significant decrease in the number of invisible TVA neurons which were infected by SADΔG-EGFP(EnvA) virus when TVA and tRFP expression was amplified using the gal4-UAS system (Fig. 1*G*). When animals were electroporated with CMV::TVA/tRFP and injected with SADΔG-EGFP(EnvA) virus 4 d later, an average of 85% of EGFP^+^ neurons per animal lacked detectable tRFP expression. In contrast, electroporating tadpoles with CMV::gal4 or VGAT::gal4 along with UAS::TVA and UAS::tRFP, resulted in only 28% of EGFP^+^ neurons which lacked detectable tRFP (*p* < 0.0001_c_; Table 1). In addition, these data demonstrate that different promoters can be used to target infection with SADΔG-EGFP(EnvA) showing its utility to target genetically defined neuronal populations.

To test whether targeting electroporation to a few cells per tectum could reduce the number of invisible TVA neurons, we electroporated tectal cells sparsely using a micropipette (Bestman et al., 2006). For this experiment, we used CMV::TVA/tRFP because micropipette-mediated electroporation of multiple plasmids is inefficient. We electroporated three to four sites within one tectum. Four days later, we injected animals with successful targeted TVA/tRFP electroporation with SADΔG-EGFP(EnvA) virus and imaged the animals 5 d later. While the majority of animals were infected (77%, *n* = 13/17 tadpoles), they still had a number of EGFP-only cells present (Fig. 1*H*). Next, we limited electroporation even further by electroporating only one site per tectum with CMV::TVA/tRFP and screened for animals with a single tRFP^+^ cell. These tadpoles were injected with SADΔG-EGFP(EnvA) virus 4 d after electroporation. However, 4–7 d after viral injection, no infected cells were detected (*n* = 11 tadpoles). These data demonstrate that using targeted micropipette electroporation did not limit viral infection to cells with detectable tRFP expression; however, these data do show that increasing tRFP co-expression in TVA^+^ neurons decreased the proportion of infected EGFP-only cells but did not eliminate them.

Together, these results demonstrate that recombinant rabies virus infects neurons in *Xenopus* tadpoles. Viral injection of B19G phenotypically complemented virus produces widespread infection and strong transgene expression in the vast majority of animals. Furthermore, pairing promoter-driven expression of TVA with injection of EnvA pseudotyped rabies virus can be used to express genes of interest in targeted neuronal populations in *Xenopus* tadpoles.

### Lack of presynaptic transfer of pseudotyped rabies virus

**Figure 2.**
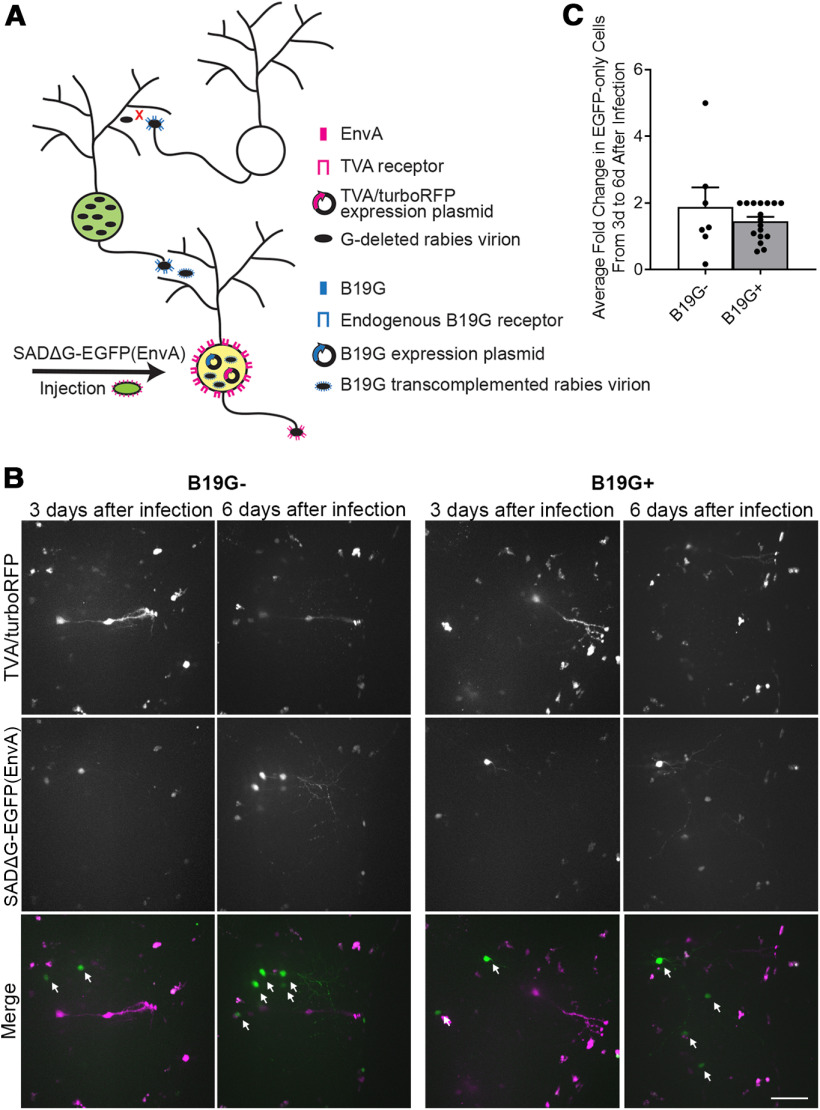
Transcomplementation with rabies glycoprotein does not result in transneuronal spread of recombinant rabies in tadpoles. ***A***, Schematic of the monosynaptic tracing strategy using SADΔG-EGFP(EnvA) virus with transcomplementation of rabies glycoprotein, B19G. Neurons co-transfected with TVA/tRFP and B19G can be directly infected by EnvA pseudotyped virus through the TVA receptor. Viral particles which bud from directly infected neurons will have B19G on their surface because B19G is provided in *trans*. In mammals, those viral particles can infect presynaptic neurons through the endogenous B19G receptor. Because presynaptically infected neurons lack B19G expression, viral particles generated in those neurons lack the glycoprotein and are not infectious, thereby prohibiting further spread. ***B***, *In vivo* time-lapse imaging of infected tectal neurons from 3–6 d following injection of SADΔG-EGFP(EnvA) virus in the presence or absence of B19G. One tectal lobe was transfected with TVA/tRFP (magenta) alone (left) or with TVA/tRFP and B19G (right) and then injected with SADΔG-EGFP(EnvA) virus 4 d later. At 3 and 6 d after viral injection, confocal Z-stacks through the tectal lobe were collected. Z-projections show an increase in the number of EGFP^+^ neurons without detectable tRFP (white arrows) from 3 to 6 d after viral injection in both the presence and absence of B19G. Scale bar: 50 μm. ***C***, Quantification of the average fold-change in the number of EGFP-only cells from 3 to 6 d after injection. There is a similar increase in the average number of EGFP-only cells over time in the presence (*n* = 17 tadpoles) and absence (*n* = 7 tadpoles) of B19G, suggesting a lack of local presynaptic spread of rabies virus. Data are presented as mean ± SEM overlaid with individual data points (*p* = 0.74, Mann–Whitney *U* test).

Pseudotyped recombinant rabies virus will undergo retrograde monosynaptic transfer when infected neurons are supplied with rabies glycoprotein, B19G in *trans* (Wickersham et al., 2007b). Viral particles which bud from infected neurons transcomplemented with B19G have glycoprotein on their surface and infect presynaptically connected cells (Fig. 2*A*). Following viral injection, neurons transfected with TVA/tRFP and directly infected by virus injection would be expected to express both tRFP and EGFP, while neurons infected via presynaptic transfer would express EGFP alone (Fig. 2*A*).

Since we observed EGFP-only cells in the absence of B19G expression (Fig. 1*C*,*D*), the presence of local EGFP-only cells could not be used as an indication of presynaptic infection under these conditions. We reasoned that if presynaptic transfer occurred in the presence of B19G expression, we would detect an increase in the number of EGFP^+^ neurons over time as virions spread presynaptically and presynaptic neurons began to express EGFP. We electroporated tadpoles with either TVA/tRFP alone, or both TVA/tRFP and B19G and injected SADΔG-EGFP(EnvA) virus 4 d later. We performed *in vivo* time lapse imaging at 3 and 6 d following virus injection and quantified the number of EGFP^+^ cells at both time points (Fig. 2*B*). On average, there was a 1.46-fold increase in the number of EGFP^+^ cells from 3 to 6 d in TVA/tRFP/B19G-expressing animals. Similarly, in animals which expressed only TVA/tRFP, and were thus incapable of presynaptic spread, there was a 1.88-fold increase in the number of EGFP^+^ neurons from 3 to 6 d after infection (*p* = 0.74_d_; Table 1; Fig. 2*C*). This result demonstrated that an increase in EGFP-expressing cells over time was not necessarily indicative of presynaptic transfer of virus.

Next, we looked for evidence of presynaptic infection by investigating whether EGFP^+^ neurons were visible outside of the injected tectal lobe. Following unilateral electroporation of TVA and B19G into a single tectal lobe, injection of SADΔG-EGFP(EnvA) virus into the transfected tectal lobe would be expected to produce presynaptic infection in several known presynaptic brain areas including the contralateral tectum and hindbrain (Hiramoto and Cline, 2009; Gambrill et al., 2016). However, when SADΔG-EGFP(EnvA) was injected into the transfected tectal lobe, no EGFP^+^ neurons were ever observed in known presynaptic brain regions (data not shown). While trans-synaptic infection occurs in mammalian systems within a week (Wall et al., 2010), this process may be slower in non-mammalian vertebrates reared at lower temperatures. Since survival and health of rabies infected neurons begins to diminish after approximately two weeks (Wickersham et al., 2007a), we looked for presynaptically infected neurons up to 10 d following viral injection in a subset of tadpoles, but still failed to observe EGFP^+^ neurons in presynaptic brain regions (*n* = 6 tadpoles, data not shown). Taken together, the results of these two experiments suggest that trans-synaptic infection of cells in the tadpole does not occur under the conditions we tested.

### Lack of presynaptic infection may be because of insufficient glycoprotein expression

The most likely explanations for a lack of presynaptic infection are (1) presynaptic terminals do not contain the receptor(s) necessary for viral uptake; (2) the virus is not packaged and transported appropriately; (3) synapses are too weak to mediate presynaptic infection; (4) electroporated B19G is not expressed sufficiently or in the correct place to coat budding virions; or (5) the virus is not released at the appropriate location. We ruled out the first possibility because B19G phenotypically complemented virus infects tectal neurons, demonstrating that the B19G receptor(s) exist in *Xenopus* tadpoles at this stage. We tested two of the other possibilities to understand why we did not detect transsynaptic infection and to identify strategies for improvement in the future.

There has been speculation that the extent of presynaptic spread might depend on the strength or number of synapses (Callaway, 2008). It is possible that the synaptic connections in young tadpoles might be too weak to efficiently mediate presynaptic infection. To test this, we exposed tadpoles to a visual stimulus before viral injection, which has previously been shown to increase synaptic strength (Ruthazer et al., 2006; Aizenman and Cline, 2007). We electroporated tadpoles with VGAT::gal4, UAS::TVA, UAS::tRFP, and UAS::B19G in one tectal lobe and then exposed them to visual experience (VE) for either: (1) 4 h of VE 2 d before viral injection, or (2) 12 h of VE the night before viral injection. Similar to other experiments, 64% of tadpoles (*n* = 9/14 tadpoles) provided with visual stimulus were infected by SADΔG-EGFP(EnvA) virus. However, we did not observe EGFP^+^ cells outside of the injected tectal lobe in any of the groups (data not shown) suggesting that increasing synaptic strength with these protocols is insufficient to produce presynaptic transfer of virus.

**Figure 3.**
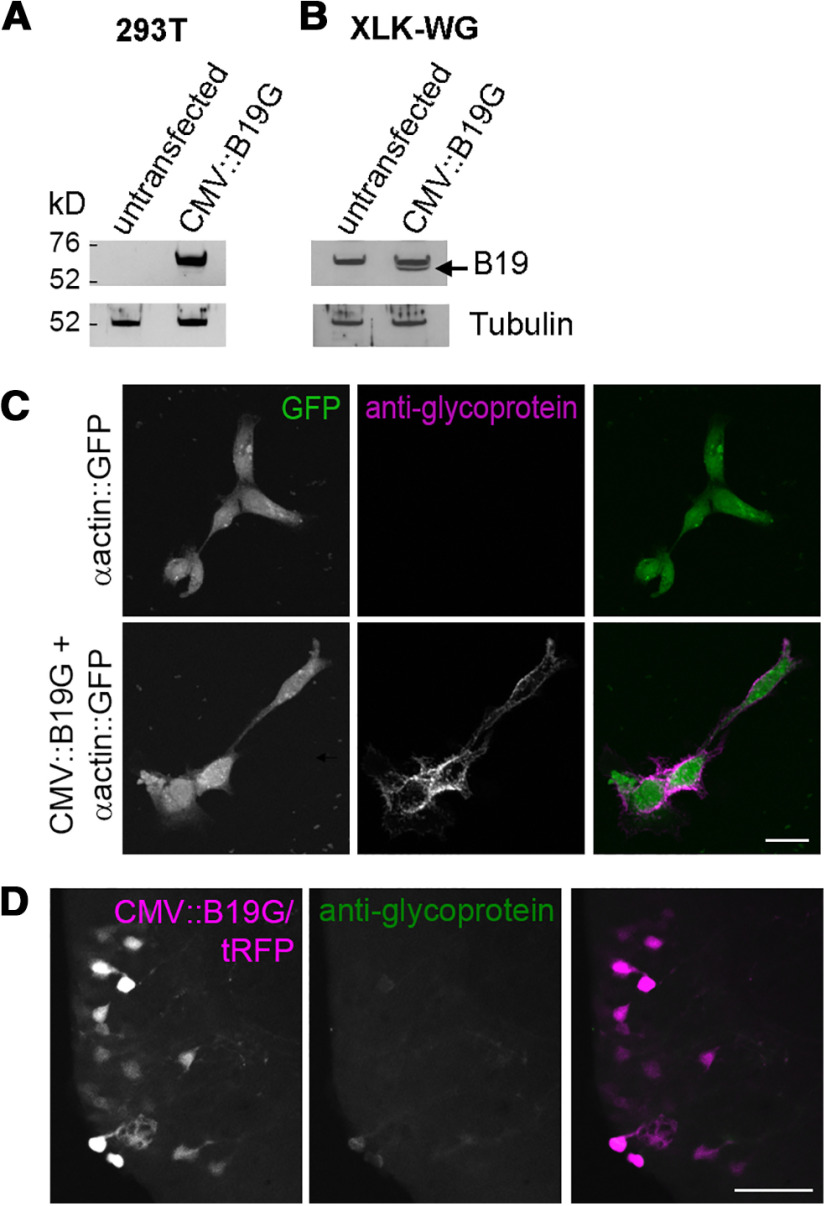
Weak expression of B19G *in vivo* may explain the lack of transneuronal spread of rabies virus. ***A***, ***B***, Rabies glycoprotein is detected in the membrane fraction of transfected mammalian and *Xenopus* cell cultures by Western blotting. 293T (***A***) and XLK-WG *Xenopus* kidney cells (***B***) were transfected with B19G and proteins were extracted 24 h later. Membrane fractions were probed for B19G expression with anti-rabies glycoprotein antibody and β-tubulin acted as a loading control. Compared with untransfected cells, specific bands of ∼70 kDa were visible in transfected cells. Specific band in transfected XLK-WG cells is denoted by an arrow (***B***). ***C***, Rabies glycoprotein is detected on the surface of *Xenopus* cells *in vitro* by immunocytochemistry. XLK-WG cells were transfected with GFP alone (top) or B19G and GFP (bottom). Confocal Z-projections of cells transfected with both B19G and GFP show surface expression of B19G by anti-rabies glycoprotein immunocytochemistry (magenta) without permeabilization. In contrast, no anti-rabies glycoprotein immunoreactivity is observed in cells transfected with GFP alone. Scale bar: 20 μm. ***D***, Expression of B19G is very weak *in vivo* in tectal neurons. Tectal neurons were electroporated with CMV::B19G/tRFP, fixed 3–4 d later, and then immunohistochemistry with anti-rabies glycoprotein was performed. Confocal Z-projection of a 40-μm tissue slice shows very weak immunoreactivity for rabies glycoprotein (green) in B19G/tRFP expressing neurons (magenta). Scale bar: 50 μm.

For presynaptic infection to occur, B19G expression is required on the cell membrane so that it coats the surface of budded viral particles. In addition, surface B19G expression increases the number of virions which bud from infected neurons (Mebatsion et al., 1996a). To test the possibility that B19G is not expressed sufficiently in transcomplemented cells, we first assessed expression of B19G *in vitro*. We transfected 293T cells with CMV::B19G and α-actin::GFP. One day after transfection, we harvested cells and did Western blottings on membrane fractions using anti-rabies glycoprotein antibody previously shown to detect B19G (Beier et al., 2019). We detected B19G in the membrane fraction of 293T cells, but no signal was found in untransfected cells (Fig. 3*A*). To test whether B19G expression occurs in frog cells, we transfected XLK-WG *Xenopus* kidney cells with CMV::B19G and α-actin::GFP, harvested cells 1 d after transfection, and did Western blottings on membrane fractions. While there was a band of similar size to rabies glycoprotein found in both transfected and untransfected cells, we observed an additional band specifically in the transfected XLK-WG cells (Fig. 3*B*). Next, we performed immunohistochemistry for the rabies glycoprotein in CMV::B19G-expressing XLK-WG cells. Cells were transfected with CMV::B19G and α-actin::GFP and fixed for immunohistochemistry 48 h later. We found that B19G was expressed on the membrane of XLK-WG cells using non-permeabilized immunohistochemistry conditions with anti-rabies glycoprotein antibody (Fig. 3*C*). In contrast, XLK-WG cells transfected with α-actin::GFP alone and imaged using identical parameters had no detectable staining with the anti-rabies glycoprotein antibody. Together, these results indicate that B19G can be expressed on the cell membrane in *Xenopus* cells *in vitro*.

Finally, we assessed the expression of B19G in tadpoles *in vivo*. We electroporated CMV::B19G/tRFP and examined expression of the glycoprotein using immunohistochemistry. Four days following electroporation, we fixed the animals, dissected and embedded their brains, sectioned them on a vibratome, and performed immunohistochemistry with anti-rabies glycoprotein antibody. Expression of B19G was either observed at low levels (Fig. 3*D*) or not at all (data not shown). When B19G signal was present, it was observed in the membrane of the apical cell body and in the proximal dendrite. By comparison, B19G expression in XLK-WG cells appeared much stronger and more uniform around the cell membrane. While we cannot rule out differences in antibody penetration in intact tissue compared with culture, these results are consistent with insufficient expression of B19G *in vivo* contributing to the lack of presynaptic spread of rabies virus.

### Retrograde labeling of neural circuits with recombinant rabies virus

**Figure 4.**
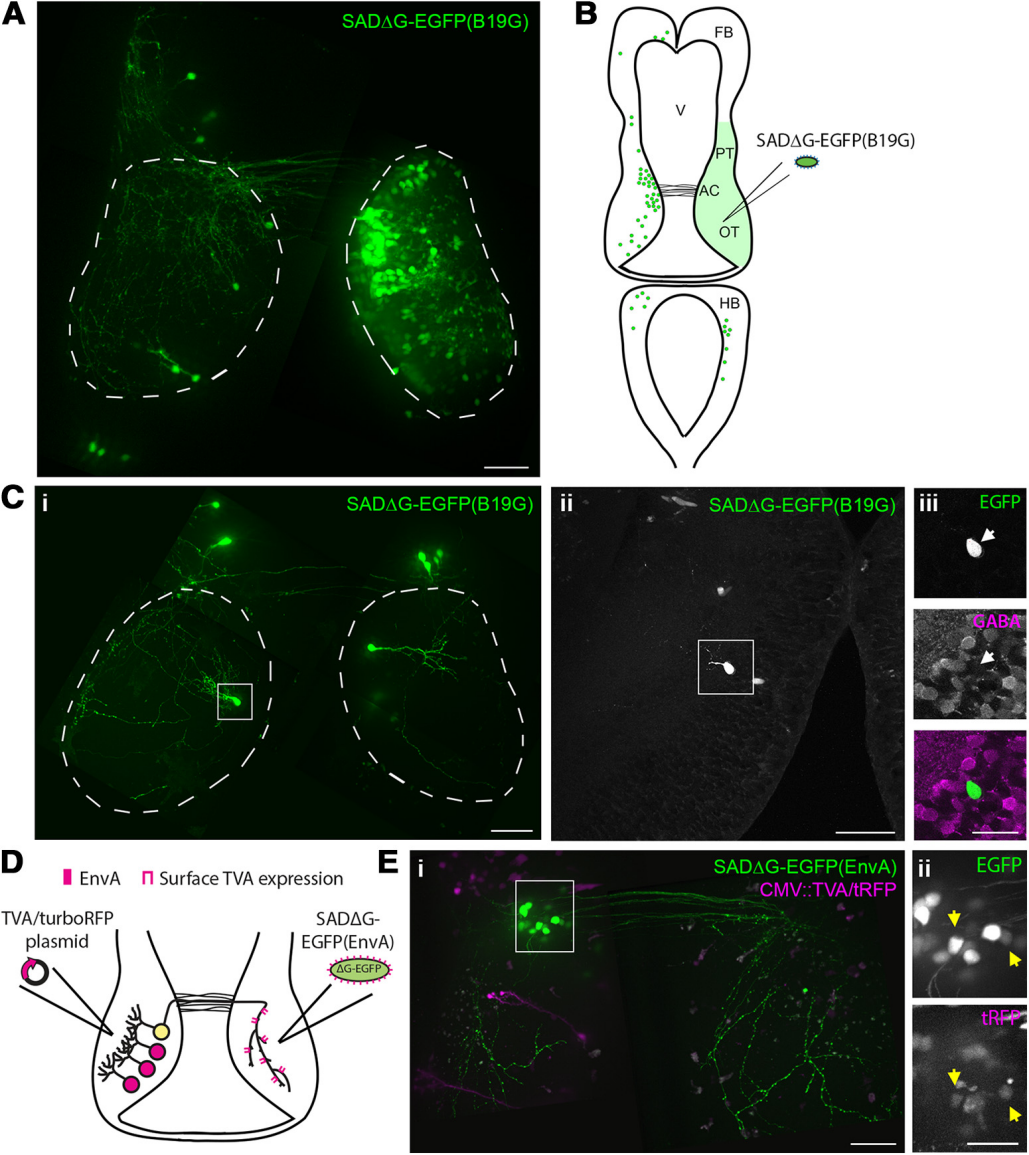
Retrograde neuronal tracing using recombinant rabies virus. ***A***, SADΔG-EGFP(B19G) virus retrogradely labels afferents to the injected target region. A montage of confocal Z-projections collected *in vivo* shows neurons infected by injection of SADΔG-EGFP(B19G) virus into the right tectal lobe. In addition to a large number of neurons expressing EGFP in the injected tectal lobe, retrogradely infected projection neurons are visible in the contralateral tectum, pretectum, and hindbrain. Tectal lobes are marked with dashed lines. Scale bar: 50 μm. ***B***, A schematic which maps neurons labeled by unilateral tectal injection of SADΔG-EGFP(B19G) virus. Neurons in several regions known to project to the optic tectum are labeled. We also observed a large number of neurons in the injected tectal lobe and ipsilateral pretectum (green shading). AC, anterior commissure; FB, forebrain; HB, hindbrain; OT, optic tectum; PT, pretectum; V, ventricle. ***C***, Retrograde viral tracing paired with immunohistochemistry reveals the cell types which project between the two tectal lobes. A montage of confocal Z-projections collected *in vivo* following injection of SADΔG-EGFP(B19G) virus into the right tectal lobe shows retrograde tracing of one intertectal neuron (boxed in left tectal lobe; ***i***). Following fixation and tissue sectioning, immunohistochemistry was performed with an anti-GABA antibody to label inhibitory neurons. The EGFP^+^ neuron imaged *in vivo* (***i***) could be identified in fixed tissue slice (***ii***) and was GABA-negative (***iii***, magenta), suggesting that it is excitatory. Scale bars: 50 μm (***i***, ***ii***) and 25 μm (***iii***). ***D***, Schematic showing retrograde tracing strategy used in ***E***. The left tectal lobe is electroporated with TVA/tRFP and 4 d later, SADΔG-EGFP(EnvA) virus is injected into the right tectal lobe. Expression of TVA on the surface of intertectal axons mediates viral infection of intertectal neurons in the left hemisphere. ***E***, SADΔG-EGFP(EnvA) virus can be used to retrogradely trace neurons defined by anatomic location and axonal projections using promoter-driven expression of TVA. A montage of confocal Z-projections collected *in vivo* (***i***) demonstrate retrograde tracing of TVA-expressing neurons in the left tectal lobe (magenta) following injection of SADΔG-EGFP(EnvA) virus into the right tectal lobe. Retrogradely infected neurons (boxed in **i**) are shown at higher magnification (***ii***) and cells co-expressing EGFP and tRFP are marked by yellow arrows. Scale bars: 50 μm (***i***) and 25 μm (***ii***).

In mammals, recombinant rabies virus can be used as a retrograde tracer since it infects at the axon terminal and is retrogradely transported to the cell soma. We tested the utility of using recombinant rabies virus as a retrograde tracer in *Xenopus* tadpoles. As demonstrated in Figure 1*B*, unilateral injections of SADΔG-EGFP(B19G) virus into the optic tectum transduced local axon terminals and yielded robust labeling at the injection site. In 43% of the infected animals (*n* = 15/35 tadpoles), we also observed retrogradely infected EGFP^+^ cells in other brain regions which project to the optic tectum (Fig. 4*A*). From these data we generated a schematic of neurons which were retrogradely labeled by unilateral tectal injection of SADΔG-EGFP(B19G) virus (Fig. 4*B*). We found retrograde labeling of neurons in regions known to project to the optic tectum including the contralateral optic tectal lobe, hindbrain, pretectum and forebrain, as well as the ipsilateral hindbrain. EGFP^+^ cells were also present in very high numbers in the ipsilateral pretectum making them difficult to count and were not included in the schematic. Retinal ganglion cells are known to be a primary source of input to the optic tectum, but no EGFP was observed in the optic chiasm and the eye was not examined in these experiments. It is possible that viral injections were made too deep to label the superficially located retinal ganglion cell axons, or that rabies poorly infects the axon terminals of some cell types in the tadpole. Nonetheless, we found robust infection in several known presynaptic areas demonstrating that rabies can transport between brain regions and act as a retrograde tracer in tadpoles.

Combining retrograde tracing with immunohistochemistry can provide information about the cell types which project to a target region of interest. For example, neurons which project between the tectal hemispheres are consistently labeled by injection of SADΔG-EGFP(B19G) virus. The development and function of intertectal inputs in tadpoles has only recently begun to be studied (Gambrill et al., 2016, 2018). Whether intertectal inputs are excitatory and/or inhibitory will have a large impact on how they contribute to the function of the tectal circuit. To ascertain whether intertectal neurons are excitatory or inhibitory, we performed live imaging of tadpoles 7 d after injection of SADΔG-EGFP(B19G) virus into the right tectal lobe to screen for infection, and then fixed infected tadpoles and performed immunohistochemistry on brain sections with an anti-GABA antibody (Fig. 4*C*). At this developmental stage, GABA immunohistochemistry can be used to determine whether neurons are excitatory or inhibitory because GABA immuno-negative neurons are positive for the excitatory neuronal marker CaMKII (Miraucourt et al., 2012). Using this strategy, the ratio of excitatory to inhibitory intertectal neurons was found to be 3:1 (Gambrill et al., 2016), closely matching the overall proportion of these neuronal types in the optic tectum at this stage (Miraucourt et al., 2012). This experiment demonstrates the utility of retrograde rabies infection of afferent neurons in the tadpole to explore questions of circuit composition.

A complementary strategy to the one described above is to use cell type-specific expression of EnvA to control the cell types which become retrogradely infected with pseudotyped rabies (Dohaku et al., 2019). We assessed whether retrograde tracing with pseudotyped rabies virus can be combined with promoter-driven expression of TVA in distant afferent neurons in the tadpole. We electroporated the left tectal lobe with TVA/tRFP driven by the CMV or VGAT promoter and then injected SADΔG-EGFP(EnvA) virus in the left or right tectal lobe 4 d later. As observed previously (Fig. 1), 59% of tadpoles (*n* = 16/27 tadpoles) injected with virus in the transfected tectal lobe had EGFP^+^ cells as a result of local viral transduction. We also found that 16% of tadpoles (*n* = 5/32 tadpoles) injected with SADΔG-EGFP(EnvA) virus in the tectal lobe contralateral to TVA/tRFP transfection had EGFP^+^ cells in the transfected tectal lobe (Fig. 4*D*,*E*). This result suggests that electroporated TVA is expressed on the afferent axons of transfected neurons and SADΔG-EGFP(EnvA) virus can infect neurons via those axons when injected into target areas. In contrast, SADΔG-EGFP(EnvA) virus injection into the transfected right tectal lobe never resulted in EGFP^+^ cells in the untransfected left tectal lobe (*n* = 0/27 tadpoles). This demonstrates that retrograde infection with SADΔG-EGFP(EnvA) required axonal expression of TVA.

Together, these experiments demonstrate that recombinant rabies virus can be used to express genes of interest in the *Xenopus* tadpole. SADΔG-EGFP(B19G) retrogradely infects both excitatory and inhibitory neurons in several brain regions. SADΔG-EGFP(EnvA) can be paired with promoter-driven expression of TVA to label targeted populations of neurons defined by cell body location and axon projection. These viral variants provide the flexibility to identify and manipulate gene expression in projection neurons in both a pan-neuronal and cell type-specific manner.

## Discussion

Here we demonstrate that recombinant rabies virus infects neurons in the *Xenopus* tadpole brain. B19G phenotypically complemented virus infects neurons via its endogenous receptor, while EnvA pseudotyped virus infects subpopulations of neurons by targeted expression of the TVA receptor. Both viruses resulted in infection in the majority of injected tadpoles between developmental stages 44–48. They produced robust transgene expression in the cell body and, sometimes, EGFP expression was bright throughout the cellular processes as well. While we do not have evidence of transneuronal spread of rabies in the tadpole, the use of rabies in *Xenopus* to combine retrograde labeling from specific axon projection sites with transgene expression will facilitate exciting new research into mesoscale connectomics and the function of neural circuits in a tractable model system (Zeng, 2018).

### Virus-mediated gene expression in non-mammalian vertebrates

Virus-mediated gene expression in non-mammalian vertebrates has had mixed results. AAV and lentivirus, which have been used with great success in mammals (Nassi et al., 2015; Parr-Brownlie et al., 2015; Haery et al., 2019), infect *Xenopus* and zebrafish either inconsistently or not at all (Zhu et al., 2009; Yamaguchi et al., 2018). *Xenopus* express two homologues of the Cosackie and adenovirus receptor (CAR) rendering them susceptible to infection by adenovirus and adenovirus-mediated expression of EGFP lasts for at least 10 d in non-neuronal tissue (Kawakami et al., 2006; Dutton et al., 2009). However, tadpoles must be maintained at increased temperature immediately following viral injection for infection to occur and it is not currently known whether neurons in the central nervous system can be infected by adenovirus. Vaccinia is a DNA virus with large packaging capacity that widely infects tadpole neurons when injected into the brain ventricle and can be targeted to specific brain regions when directly injected into brain tissue (Wu et al., 1995). Vaccinia produces robust transgene expression at normal rearing temperature; however, transgene expression is transient, decreasing over 10 d after infection. The versatility of vaccinia is limited because it cannot be restricted to specific cell types using promoters. VSV, like rabies, is an enveloped negative-sense RNA virus (Nassi et al., 2015) that can reportedly infect neurons in both *Xenopus* and zebrafish (Mundell et al., 2015; Yamaguchi et al., 2018). In zebrafish, VSV encoding the VSV glycoprotein VSV(VSV-G) infects neurons and undergoes anterograde transneuronal spread, while VSV encoding the rabies glycoprotein VSV(RABV-G) infects neurons and undergoes retrograde transneuronal spread (Mundell et al., 2015; Ma et al., 2019). Yamaguchi et al. (2018) found that direct injection of VSV(VSV-G) and VSV(RABV-G) into the brains of *Xenopus* frogs and tadpoles infects neurons but does not spread transneuronally. In contrast, Mundell et al. (2015) reported anterograde transneuronal transfer of VSV(VSV-G) from the eye to the brain in *Xenopus* tadpoles, but the data were not shown. It is not clear what accounts for the difference between these findings, but both the age of the animals and the site of viral injection differed and could contribute to the variability in the results. These studies demonstrate that while viral tools for *Xenopus* are available, rabies is an attractive addition to the viral toolbox.

### Lack of presynaptic viral spread in *Xenopus*

We were unable to detect retrograde transneuronal spread of pseudotyped rabies in *Xenopus* tadpoles. Similarly, transneuronal transfer was not observed in adult *Xenopus* frogs injected with recombinant VSV encoding the rabies glycoprotein (Yamaguchi et al., 2018). Transneuronal transfer of rabies requires replication and transport of rabies virions, budding of rabies virions from the postsynaptic cell, expression of glycoprotein on the surface of budded virions, and presynaptic expression of the rabies glycoprotein receptor. We observed infection with SADΔG-EGFP(B19G) demonstrating that the rabies glycoprotein receptor is expressed in *Xenopus* tadpole brain. Given the wide range of neurons which can be infected by rabies in mammals, the glycoprotein receptor is thought to be ubiquitous (Kelly and Strick, 2000). NCAM, P75ntr, and mGluR2 have been identified as potential receptors for rabies glycoprotein in the brain (Thoulouze et al., 1998; Tuffereau et al., 1998; Wang et al., 2018), although P75ntr is not required for infection (Tuffereau et al., 2007). All three are also expressed in the *Xenopus* brain to mediate infection with SADΔG-EGFP(B19G) in tadpoles (Session et al., 2016). Following infection at the axon terminal, rabies virions are transported to the cell body for replication, and then spread to dendrites for release. Inefficient viral replication or transport of rabies virions out to the dendrites could contribute to a lack of presynaptic infection. Based on the level of EGFP expression from the virus, replication of rabies virus may be diminished in our system compared with mammals. In mammalian systems, neurons infected with recombinant rabies invariably express very high levels of fluorescent protein making the entire neuron visible (Wickersham et al., 2007a; Marshel et al., 2010). We sometimes observed beautiful EGFP labeling throughout entire neuronal arbors, but often, only cell bodies were labeled by EGFP. This could be a result of inefficient viral replication at the colder temperatures (∼20°C) required for rearing *Xenopus*. Future work using recombinant rabies in *Xenopus* should take this variable into account. Manipulations that require labeling the entire neuronal arbor or high expression levels of genes of interest may be accomplished by incorporating amplification mechanisms, such as gal4-UAS or Cre-recombinase. Increasing the rearing temperature in *Xenopus* has been shown to facilitate infection with adenovirus (Dutton et al., 2009) and rearing zebrafish between 34°C and 35.5°C was required for transneuronal transfer of SADΔG-EGFP(EnvA) (Dohaku et al., 2019). We found that a modest, short term increase in rearing temperature did not increase the percentage of animals infected by rabies virus, however additional experiments exploring varying the rearing temperature of tadpoles might improve transgene expression or transneuronal transfer.

Two likely explanations for a lack of transneuronal infection in *Xenopus* tadpole brain are insufficient expression of rabies glycoprotein and/or problems with viral budding. We did not assess whether the virus buds appropriately from *Xenopus* neurons. Two possible ways to investigate this in the future are to infect *Xenopus* cells *in vitro* and then measure viral proteins in collected culture media or to perform electron microscopy of infected tectal neurons *in vivo*. We assessed the expression of rabies glycoprotein in *Xenopus* neurons both *in vitro* and *in vivo*. Exogenously expressed glycoprotein was detected on the membrane of XLK-WG cells *in vitro* by Western blotting and immunocytochemistry. However, there was very little expression detected by immunohistochemistry in tectal neurons following electroporation *in vivo*. Yamaguchi et al. (2018) demonstrated that B19G incorporated into the VSV genome was also insufficient to produce transneuronal transfer, suggesting that the difficulty of expressing B19G *in vivo* in *Xenopus* is not specific to electroporation. While viral particles lacking the glycoprotein are capable of budding from cells, presence of the glycoprotein increases budding of viral particles by 30-fold (Mebatsion et al., 1996a). Therefore, improving glycoprotein expression would not only facilitate binding of viral particles to the presynaptic terminal during transneuronal spread, but it would also improve budding of viral particles from infected postsynaptic neurons. There are several possibilities which could explain poor glycoprotein expression and ways that it could be improved. Rabies glycoprotein has multiple glycosylation sites and at least one of them needs to be glycosylated for expression of glycoprotein on the membrane (Dietzschold, 1977; Conzelmann et al., 1990; Shakin-Eshleman et al., 1992). Previously, injection of rabies glycoprotein RNA into *Xenopus* oocytes was found to produce an unglycosylated protein product (Wunner et al., 1980). Whether rabies glycoprotein is glycosylated in *Xenopus in vivo* remains to be investigated. Expressing the glycoprotein from a different strain of rabies could be a way to achieve transneuronal tracing in *Xenopus*. The pathogenicity of different rabies strains is determined, in large part, by their glycoproteins. The glycoproteins are expressed at different levels on the cell surface and contribute to different rates of viral replication and spread (Dietzschold et al., 2008). Furthermore, the distribution of presynaptic neurons labeled following viral injection depends on which rabies strain the glycoprotein is taken from, suggesting that the tropism may vary between different glycoproteins (Yan et al., 2002). The challenge virus standard (CVS) strain of rabies has also been used extensively for transneuronal tracing (Astic et al., 1993; Ugolini, 1995; Kelly and Strick, 2000; Reardon et al., 2016). In mice, G-deleted CVS-N2c virus phenotypically complemented with N2c-G or G-deleted SAD B19 virus pseudotyped with N2c-G infect ∼10-fold more presynaptic neurons compared with SADΔG-EGFP(B19G) (Reardon et al., 2016; Zhu et al., 2020). A chimeric glycoprotein containing the cytoplasmic domain of the parent SAD B19 glycoprotein and the extracellular domain of the glycoprotein from the Pasteur virus (PV) strain of rabies also resulted in higher presynaptic infection in mouse brain (Kim et al., 2016). The efficacy of presynaptic infection increased even further when the chimeric glycoprotein was codon optimized, yielding 20-fold more presynaptic neurons compared with non-optimized B19G (Kim et al., 2016). Optimizing codon usage for the desired species has been shown to increase glycoprotein expression by 2-fold (CVS-N2cG) (Wirblich and Schnell, 2011). Using glycoproteins from different strains which are codon optimized for *Xenopus* has the potential to improve glycoprotein expression and transneuronal spread.

### Mesoscale brain circuitry analysis with rabies virus

Neuroanatomical brain connectivity studies have helped to uncover the structure of individual neurons and neuronal circuits, while expression of transgenes in neurons allows for their visualization and manipulation (Vercelli et al., 2000; Nassi et al., 2015; Luo et al., 2018; Zeng, 2018). These techniques have contributed greatly to our understanding of the brain. Rabies combines these tools into a single reagent capable of retrogradely labeling neurons based on axonal projections and simultaneously expressing transgenes to visualize or manipulate neuronal activity (Osakada et al., 2011; Nassi et al., 2015). The widespread adoption of rabies for mesoscale circuit analysis has led to the creation of a plethora of viral variants which could be used in *Xenopus*.

Cells infected with recombinant rabies virus engineered to express fluorescent proteins and phenotypically complemented with B19G are intensely labeled as a result of viral replication. Combining rabies retrograde tracing with *post hoc* immunohistochemistry reveals the cell types which contribute to neural circuits and suggests that application of rabies virus to study mesoscale connectomics in *Xenopus* will generate new insights into circuit components and circuit function. For instance, we recently investigated the development and function of a direct intertectal projection in tadpoles. Using rabies virus injection followed by *post hoc* immunohistochemistry, we found that both excitatory and inhibitory tectal neurons contribute to intertectal communication, which has major implications for how this neural circuit contributes to tectal function (Gambrill et al., 2016). We found that injection of SADΔG-EGFP(B19G) directly into the brain ventricle resulted in widespread infection near the injection site. This strategy could be exploited to infect neurons and express genes of interest when retrograde tracing from a specific target is not needed. Rabies virus variants are available which drive expression of many fluorescent proteins including GFP, DsRed, mCherry, and BFP (Osakada et al., 2011). In principle, intersectional analysis using simultaneous injection of rabies variants expressing different color fluorescent proteins into different target areas could be used to assess the distribution of neurons projecting to those different targets. In addition, by identifying doubly-labeled or triply-labeled neurons, the degree to which single neurons send axon collaterals to multiple targets can be evaluated. Pseudotyping rabies with the VSV glycoprotein converts it into an anterograde tracer (Wickersham et al., 2013). Combining injection of anterograde and retrograde rabies variants expressing different color fluorescent proteins could simultaneously label the neurons projecting to that brain region and the axonal projections from that brain region to its targets, providing an additional level of detail about relationships between neuronal inputs and outputs. Rabies variants which express multiple transgenes in a single virus can be used to simultaneously label different cellular compartments such as cytoplasmic GFP to visualize axonal arbors and synaptophysin-RFP to label presynaptic profiles (Wickersham et al., 2013). This would allow for visualization of rapid changes in synaptic connectivity during plasticity. These experiments could be specialized further by cell type-specific infection.

Rabies virus pseudotyped with EnvA is a powerful intersectional approach which can be used to label neurons in both an anatomically and genetically defined manner. As proof of principle, we expressed TVA in one tectal lobe and then injected EnvA pseudotyped rabies into the other tectal lobe and successfully retrogradely infected only intertectal neurons expressing TVA. In our system, VGAT promoter-driven expression of TVA is biased toward inhibitory neurons but is not cell type specific. This lack of specificity likely reflects cellular fate specification in the developing tadpole brain. Neurons at the stages of development used in our study can dynamically switch neurotransmitter expression (Borodinsky et al., 2004; Dulcis et al., 2013; Li et al., 2020). Furthermore, the inhibitory neuron markers GABA and GAD67, and the excitatory neuron marker CaMKII, are regulated by visual activity in the tadpole optic tectum (Miraucourt et al., 2012; Shen et al., 2014). In addition, single cell RT-PCR showed that individual tectal neurons express transcripts of both excitatory and inhibitory markers (Cline lab, unpublished observations). Nevertheless, cell type-specific expression plasmids, for instance Sox2 in neural progenitor cells (Bestman et al., 2012), do function in the *Xenopus* brain. Therefore, our results suggest that an intersectional strategy using cell type-specific expression of TVA is possible in principle, based on the specificity of available promoters.

Driving TVA expression using cell type-specific promoters would permit the visualization of neurons triply-defined by cell body location, genetics, and axon projection. Neuronal cell types are defined by a combination of features including morphology, location, projection pattern, expression of genetic markers, and physiological properties (Ecker et al., 2017). Describing cell types based on these features is essential to understanding their function within the brain. The ability of rabies to label cells defined by a combination of features and simultaneously drive expression of calcium indicators to visualize neuronal activity or optogenetic tools to activate or deactivate them, can contribute a great deal to our understanding of neuronal cell types and their functions (Osakada et al., 2011). In addition to EnvA/TVA, more flexibility in cell type-specific labeling is possible using EnvB/TVB and EnvE/TVE pairs for targeting infection (Choi et al., 2010; Suzuki et al., 2019). When these viruses drive expression of different color fluorescent proteins, they can be combined in a multiplex strategy in which one performs retrograde infection of different Env( X) viruses from a single target location and labels different genetically defined subpopulations of input neurons through specific TV( X) expression. The versatility of these tools enables a variety of experiments to analyze and manipulate mesoscale connectivity in *Xenopus*, a system that has contributed to understanding fundamental principles of brain circuit development and plasticity, hormonal regulation of metamorphosis, and regeneration.

**Table 1:** Statistical table

	Data structure	Type of test	*p* value	Sample size (*n* = tadpoles)
a		Fisher’s exact test	0.37	22°C *n* = 12 28°C *n* = 11
b		Fisher’s exact test	0.63	CMV::gal4 *n* = 4 VGAT::gal4 *n* = 21
c	Not normally distributed	Mann–Whitney *U* test	<0.0001	gal4-UAS amplification *n* = 9 No gal4-UAS amplification *n* = 14
d	Not normally distributed	Mann–Whitney *U* test	0.74	B19G^+^ *n* = 17 B19G^–^ *n* = 11

For each statistical test run in the study, the data structure, statistical test, *p* value, and sample size are listed.

## Synthesis

Reviewing Editor: Alfonso Represa, INSERM Decisions are customarily a result of the Reviewing Editor and the peer reviewers coming together and discussing their recommendations until a consensus is reached. When revisions are invited, a fact-based synthesis statement explaining their decision and outlining what is needed to prepare a revision will be listed below. The following reviewer(s) agreed to reveal their identity: Ayako Yamaguchi, Constance Cepko.

In this paper, the authors systematically investigated whether rabies virus can be used to transduce neurons and express transgenes in Xenopus laevis tadpoles. the authors show that RABVdG (RABVG) and RABVdG (EnvA) can transduce neurons and express transgenes in Xenopus tadpoles. They suggest that a rabies viral vector is a valuable tool to study neural circuits in Xenopus. However, more evidence is need to support the claim that a recombinant rabies vector can be used to express transgenes in cells triply defined by cell body location, genetics, and axon projections.

Major points:

1. In one of the experiments, TVA/tRFP expression was driven by two different promoters (the CMV or VGAT), but the reported results seem to combine the transduction efficiency using both promoters, and the difference in the transduction patterns was not reported. Did the authors confirm that VGAT-driven TVA expression results in selective transduction of GABA+ neurons using immunohistochemistry?

2. in line 49-50, the authors mentioned ‘VSV transduction is inefficient’ and cited ‘Yamaguchi et al., 2018’ as a reference, however, the conclusion of Yamaguchi et al., 2018 is ‘We found that VSV is a quick, efficient, and robust viral vector that can be used reliably to transduce neurons of adult X. laevis...’.

3. line 106-107, the authors should double check the titer of SAD-dG? 10^8-10^9 TU/ul or TU/ml?

4. line 220-222, from the 83% (22{degree sign}C) or 64% (26-28{degree sign}C) infection rates, how do the authors make the conclusion that ‘increased temperature did not impact infection rates’?

5. line 246-248, the authors mention ‘In the absence of TVA, we did not observe any EGFP+ cells’. However, in Figure 1E, there is one dim green cell. What are the titers of the SAD-dG(EnvA) stock on TVA-expressing and TVA-negative cell lines? This is the best way to assess infections of non-TVA+ cells.

6. line 256-269, both VGAT::gal4 and CMV::gal4 are mentioned in the text, but only VGAT::gal4 data are shown in Figure 1F. The authors should clarify how the experiments are conducted.

7. line 313-316, based on the text, the neurons were not electroporated with both TVA/tRFP and B19G,. Why do the authors expect to see transsynaptic spread of RABVdG? This should be clarified.

8. line 333-334, the anti-rabies antibody (Millipore, MAD8727) is a monoclonal antibody that recognizes the epitope of the glycoprotein in the vaccine strain ERA. First, the authors should cite a reference to support the choice of this antibody to recognize B19G. Second, based on the text, ’electroporated the tadpole optic tectum with CMV::TVA/turboRFP and injected SADΔG-EGFP(EnvA) virus 4 days later’, why do the authors expect to detect RABVG here? Third, since Figure 3A shows positive immunostaining signals using anti-RABVG antibody, the authors should double check whether the SADΔG-EGFP(EnvA) stock is contaminated by RABVG. This is a known problem in stocks that originate with a RABVG coated RABV, as is the case here. See Calloway’s publications on this topic.

9. Figure 3D, the authors should show the immunostaining data of 293T and XLK-WG without CMV::B19G transfection.

10. Figure 4E, why are there many dim magenta cells and few bright GFP+ cells in the right tectal lobe? How confined are the plasmid electroporations and virus injection?

Other points:

12. line 106-107, the authors should double check the titer of SAD-dG? 10^8-10^9 TU/ul or TU/ml?

11. in line 119-122, the authors should provide more details of the plasmid construct. for example, is TVA800 or TVA950 used? How is bidirectional plasmid CMV::TVA/tRFP designed?

13. line 192-198, ‘pseudotyped’ is not appropriate to describe SAD-dG(RABVG)? ‘Pseudotyping’ means producing viruses in combination with a foreign glycoprotein. Therefore, line 197, ‘SADΔG-EGFP pseudotyped with its native glycoprotein’ is illogical.

14. line 220-222, from the 83% (22{degree sign}C) or 64% (26-28{degree sign}C) infection rates, how do the authors make the conclusion that ‘increased temperature did not impact infection rates’?

15. line 246-248, the authors mention ‘In the absence of TVA, we did not observe any EGFP+ cells’. However, in Figure 1E, there is one dim green cell. What are the titers of the SAD-dG(EnvA) stock on TVA-expressing and TVA-negative cell lines? This is the best way to assess infections of non-TVA+ cells.

16. line 256-269, both VGAT::gal4 and CMV::gal4 are mentioned in the text, but only VGAT::gal4 data are shown in Figure 1F. The authors should clarify how the experiments are conducted.

17. line 310, is it possible that the transsynaptic infection takes longer than 6 days, especially for a poikilotherm?

18. line 317, insert “data not shown” after “no EGFP+ neurons were ever observed in know presynaptic brain regions"

18. line 371, insert “data not shown” after “or not at all"

19. line 450, insert “in non-neuronal tissue” after “10 days"

## Author Response

Dear Dr. Represa,

Thank you for reviewing our paper “Application of Recombinant Rabies Virus to Xenopus Tadpole Brain”. We thank the reviewers for their detailed reading of our manuscript. We have addressed all of the concerns raised by the reviewers. Our response to each reviewer comment is below.

Major points:

1. In one of the experiments, TVA/tRFP expression was driven by two different promoters (the CMV or VGAT), but the reported results seem to combine the transduction efficiency using both promoters, and the difference in the transduction patterns was not reported. Did the authors confirm that VGAT-driven TVA expression results in selective transduction of GABA+ neurons using immunohistochemistry?

We have now reported the transduction efficiency for the two promoters separately and used a Fisher’s exact test to show that the proportion of animals infected was similar between the two promoters (lines 278-279).

Use of the VGAT promoter in tadpole to bias expression toward inhibitory neurons has been published in detail (He et al 2016). More of this previously published characterization has been added to the Results section (lines 273-275) and summarized here. The proportion of excitatory:inhibitory neurons in the tectum is 70:30. The VGAT promoter increases expression in inhibitory cells so the population is now 50:50 E:I as opposed to 70:30 E:I. While this is not a pure inhibitory neuron population, we used these two promoters, CMV and VGAT, as proof of principle that multiple promoters can be used to express TVA.

2. in line 49-50, the authors mentioned ‘VSV transduction is inefficient’ and cited ‘Yamaguchi et al., 2018’ as a reference, however, the conclusion of Yamaguchi et al., 2018 is ‘We found that VSV is a quick, efficient, and robust viral vector that can be used reliably to transduce neurons of adult X. laevis...’.

This statement is based on personal communication following the publication of Yamaguchi et al 2018. We have updated the citation to reflect this personal communication (line 50).

3. line 106-107, the authors should double check the titer of SAD-dG? 10^8-10^9 TU/ul or TU/ml?

The reviewers are correct that the titer is TU/mL. We have corrected this error (line 109).

4. line 220-222, from the 83% (22C) or 64% (26-28C) infection rates, how do the authors make the conclusion that ‘increased temperature did not impact infection rates’?

We used a Fisher’s exact test to compare the proportions of infected and uninfected cells when varying temperature (p=0.37). This has been added to the text (line 231) and statistical table.

5. line 246-248, the authors mention ‘In the absence of TVA, we did not observe any EGFP+ cells’. However, in Figure 1E, there is one dim green cell. What are the titers of the SAD-dG(EnvA) stock on TVA-expressing and TVA-negative cell lines? This is the best way to assess infections of non-TVA+ cells.

We have added text to the Results section (lines 256-259) to reflect the following: For these experiments, the same aliquot of virus was used to infect TVA+ and TVA- tadpoles. TVA+ and TVA- images were collected with identical imaging parameters.

Because images were collected in vivo, there is occasionally some background fluorescence which can be detected, particularly from the skin surface. The fluorescent speck that the reviewers are referring to is not in the cell body layer.

6. line 256-269, both VGAT::gal4 and CMV::gal4 are mentioned in the text, but only VGAT::gal4 data are shown in Figure 1F. The authors should clarify how the experiments are conducted.

Both VGAT::gal4 and CMV::gal4 data are included in Fig 1G. A sample using VGAT::gal4 was chosen as a representative image for Fig 1F.

7. line 313-316, based on the text, the neurons were not electroporated with both TVA/tRFP and B19G,. Why do the authors expect to see transsynaptic spread of RABVdG? This should be clarified.

We thank the reviewers for catching this error. In these experiments, the animals were electroporated with both TVA/tRFP AND B19G. The text has been updated (line 331).

8. line 333-334, the anti-rabies antibody (Millipore, MAD8727) is a monoclonal antibody that recognizes the epitope of the glycoprotein in the vaccine strain ERA. First, the authors should cite a reference to support the choice of this antibody to recognize B19G. Second, based on the text, ‘electroporated the tadpole optic tectum with CMV::TVA/turboRFP and injected SADΔG-EGFP(EnvA) virus 4 days later’, why do the authors expect to detect RABVG here? Third, since Figure 3A shows positive immunostaining signals using anti-RABVG antibody, the authors should double check whether the SADΔG-EGFP(EnvA) stock is contaminated by RABVG. This is a known problem in stocks that originate with a RABVG coated RABV, as is the case here. See Calloway’s publications on this topic.

MAB8727 has previously been used to detect B19G. This reference (Beier et al 2019, Cell Reports) has been added to the text (line 371).

We have decided to remove the data showing detection of G-deleted rabies with anti-glycoprotein antibody from Figure 3. This observation has been made previously in the Callaway lab (personal communication) but has not been published. We attribute this to the antibody not being specific to glycoprotein because the immunogen was whole virus. While we cannot completely rule out contamination, we do not believe binding of the anti-glycoprotein antibody to the G-deleted virus is a result of contamination. If that were the case, the level of contamination required to obtain the immunohistochemical staining we observed would be substantial. One would predict that level of contamination would have resulted in neuronal infection with SADΔG-EGFP(EnvA) in the absence of TVA, and we did not observe this. However, we do not have a detailed characterization of this antibody which we can provide in the manuscript and we feel it would be best to remove the data from Figure 3. The removal of this data does not change our interpretation from Figure 3 that insufficient glycoprotein expression in transcomplemented neurons may be responsible for the lack of presynaptic infection which we observed. We discuss several other possible explanations for this lack of presynaptic infection in the Results and Discussion sections. With the removal of this data from Figure 3, our Results and Discussion sections now leave open the possibility that rabies virions fail to spread throughout the dendrites where they can bud from the postsynaptic neuron thereby preventing presynaptic infection.

9. Figure 3D, the authors should show the immunostaining data of 293T and XLK-WG without CMV::B19G transfection.

Our new Figure 3C includes immunostaining data from transfected and untransfected XLK-WG cells. The data show that immunostaining with the anti-rabies glycoprotein antibody is specific to transfected cells. We have made changes to the Methods (lines 176-184) and Results (lines 381-382) sections to reflect this new data.

10. Figure 4E, why are there many dim magenta cells and few bright GFP+ cells in the right tectal lobe? How confined are the plasmid electroporations and virus injection?

We have added some more detail to the Methods to address this question. Electroporations transfect cells throughout the rostrocaudal extent of the tectum (line 130). The number of cells transfected by electroporation varies with concentration and size of the plasmid DNA and the electroporation parameters. Virus was always injected in the widest part of the tectal lobe to prevent leakage from the tissue into the ventricle (lines 112). Variability in the amount of virus injected into each tadpole mostly arose from differences in the size of animals (smaller animal = less virus). In the experiment shown in Fig 4E, the number and location of GFP+ cells also depended on the number and location of intertectally-projecting neurons.

Other points:

12. line 106-107, the authors should double check the titer of SAD-dG? 10^8-10^9 TU/ul or TU/ml?

See #3 above.

11. in line 119-122, the authors should provide more details of the plasmid construct. for example, is TVA800 or TVA950 used? How is bidirectional plasmid CMV::TVA/tRFP designed?

We have added more detail to the Methods section to reflect that we used TVA800 (line 117). For the bidirectional plasmid, a sCMV promoter is used to drive tRFP and a separate sCMV IE94 enhancer/promoter is used to drive TVA (line 123-124). This plasmid has been deposited in Addgene and its Addgene ID number has been added to the Methods section.

13. line 192-198, ‘pseudotyped’ is not appropriate to describe SAD-dG(RABVG)? ‘Pseudotyping’ means producing viruses in combination with a foreign glycoprotein. Therefore, line 197, ‘SADΔG-EGFP pseudotyped with its native glycoprotein’ is illogical.

We changed the wording in this section from ‘pseudotyped’ to ‘phenotypically complemented’. This is consistent with the terminology used in the studies which first generated and tested SADΔG(B19G) (Mebatsion et al 1996 and Etessemi et al 2000). We have also removed other instances where the term ’pseudotyped’ was used to describe SADΔG-EGFP(B19G) throughout the manuscript.

14. line 220-222, from the 83% (22{degree sign}C) or 64% (26-28{degree sign}C) infection rates, how do the authors make the conclusion that ‘increased temperature did not impact infection rates’?

See #4 above.

15. line 246-248, the authors mention ‘In the absence of TVA, we did not observe any EGFP+ cells’. However, in Figure 1E, there is one dim green cell. What are the titers of the SAD-dG(EnvA) stock on TVA-expressing and TVA-negative cell lines? This is the best way to assess infections of non-TVA+ cells.

See #10 above.

16. line 256-269, both VGAT::gal4 and CMV::gal4 are mentioned in the text, but only VGAT::gal4 data are shown in Figure 1F. The authors should clarify how the experiments are conducted.

See #6 above.

17. line 310, is it possible that the transsynaptic infection takes longer than 6 days, especially for a poikilotherm?

Yes, it is possible that transsynaptic infection is slower in Xenopus. In a subset of animals, we looked up to 10 days after viral injection and did not see presynaptically infected neurons. This time interval was chosen because the longer one waits after infection, the higher the chance that the cell health will be adversely affected by the rabies infection. In mammalian systems, health of infected neurons begins to decline after about 2 weeks. We have included comments on this topic to the Results section (lines 336-340).

18. line 317, insert “data not shown” after “no EGFP+ neurons were ever observed in know presynaptic brain regions”

This change has been made (line 335).

18. line 371, insert “data not shown” after “or not at all”

This change has been made (line 389).

19. line 450, insert “in non-neuronal tissue” after “10 days"

This change has been made (line 469).
